# Ongoing HIV-1 evolution and reservoir reseeding in two elite controllers with genetically diverse peripheral proviral quasispecies

**DOI:** 10.1590/0074-02760230066

**Published:** 2023-06-05

**Authors:** Suwellen Sardinha Dias de Azevedo, Fernanda Heloise Côrtes, Larissa M Villela, Brenda Hoagland, Beatriz Grinsztejn, Valdilea Gonçalvez Veloso, Mariza G Morgado, Gonzalo Bello

**Affiliations:** 1 Fundação Oswaldo Cruz-Fiocruz, Instituto Oswaldo Cruz, Laboratório de AIDS & Imunologia Molecular, Rio de Janeiro, RJ, Brasil; 2 Fundação Oswaldo Cruz-Fiocruz, Instituto Nacional de Infectologia Evandro Chagas, Rio de Janeiro, RJ, Brasil

**Keywords:** HIV-1, elite controllers, proviral reservoir, viral diversity, viral evolution

## Abstract

**BACKGROUND:**

Elite controllers (EC) are human immunodeficiency virus (HIV)-positive individuals who can maintain low viral loads for extended periods without antiretroviral therapy due to multifactorial and individual characteristics. Most have a small HIV-1 reservoir composed of identical proviral sequences maintained by clonal expansion of infected CD4^+^ T cells. However, some have a more diverse peripheral blood mononuclear cell (PBMC)-associated HIV-1 reservoir with unique sequences.

**OBJECTIVES:**

To understand the turnover dynamics of the PBMC-associated viral quasispecies in ECs with relatively diverse circulating proviral reservoirs.

**METHODS:**

We performed single genome amplification of the *env* gene at three time points during six years in two EC with high intra-host HIV DNA diversity.

**FINDINGS:**

Both EC displayed quite diverse PBMCs-associated viral quasispecies (mean *env* diversity = 1.9-4.1%) across all time-points comprising both identical proviruses that are probably clonally expanded and unique proviruses with evidence of ongoing evolution. HIV-1 *env* glycosylation pattern suggests that ancestral and evolving proviruses may display different phenotypes of resistance to broadly neutralising antibodies consistent with persistent immune pressure. Evolving viruses may progressively replace the ancestral ones or may remain as minor variants in the circulating proviral population.

**MAIN CONCLUSIONS:**

These findings support that the high intra-host HIV-1 diversity of some EC resulted from long-term persistence of archival proviruses combined with the continuous reservoir’s reseeding and low, but measurable, viral evolution despite undetectable viremia.

A few percentage (< 0.5%) of human immunodeficiency virus (HIV)-1-infected individuals, termed elite controllers (EC), maintained a sustained control of viral replication in the absence of antiretroviral therapy (ART).^1^ Most EC displayed a small and homogeneous HIV-1 reservoir mostly composed by identical proviral sequences that is maintained by the clonal expansion of infected memory/naïve CD4^+^ T cells and by the persistence of long-lived CD4^+^ T cells that were latently infected with identical proviruses around the time of viral transmission.^2^ ^-^ ^9^ Some EC, however, displayed a more heterogeneous peripheral blood mononuclear cell (PBMC)-associated HIV-1 reservoir mostly composed by unique sequences.^10^ ^,^ ^11^ ^,^ ^12^ The mechanism that maintained a relatively diverse PBMC-associated HIV-1 population in some EC is not well-defined. Such a pattern of relatively high intra-host HIV-1 diversity in some EC may be due to a process of ongoing replication and reservoir’s reseeding^12^ ^,^ ^13^ ^,^ ^14^ ^,^ ^15^ and/or may be consequence of a high genetic diversity in the transmitted viral population that persisted during the HIV-1 infection history.^16^ ^,^ ^17^ ^,^ ^18^ ^,^ ^19^

One previous study observed clear evidence of HIV-1 evolution in plasma sequences recovered from at least 40% of the EC.^14^ Other longitudinal studies confirmed that finding, but failed to detect measurable evolution of PBMCs-associated proviral sequences.^13^ ^,^ ^20^ Another study demonstrates that the HIV-infected CD4^+^ T cell pool in peripheral blood from EC largely comprised archival proviruses that appeared to be clonally expanded, while plasma viral sequences derived from recently infected CD4^+^ T cells populations from lymphoid tissue that were very rarely detected among circulating memory CD4^+^ T cells.^9^ Those findings may indicate that recirculation of recently infected cells from lymphoid tissue to peripheral blood in EC is very restricted and that ongoing replication in EC does not result in a significant reseeding of the circulating reservoir. Alternatively, it is also possible that newly HIV-infected cells recirculate from lymphoid tissue to peripheral blood in EC at a very slow rate and that reseeding of the circulating reservoir is only detectable after long-term follow-up, as observed in patients under ART.^21^

In previous studies conducted by our group, we identified some EC with quite diverse PBMC-associated proviral populations (mean *env* nucleotide diversity > 2%) and the longitudinal analysis of one of those individuals (named EEC42) over a long sampling time interval (> 10 years) demonstrates a low, but measurable, *env* divergence rate (3 x 10^-3^ substitutions/site/year).^12^ Patient EEC42, however, was one of the EC from our cohort with the highest frequency of blips (30% of viral load determinations above the detection limit of commercial assays) during follow-up. In the present study we assess the long-term (⁓ 6 years) evolutionary pattern of the *env* gene of the PBMC-associated HIV DNA reservoir in two EC (named EEC36 and PEC38) from our cohort that harbour diverse proviral quasispecies in the setting of persistent undetectable viremia or infrequent blips (< 15% of viral load determinations). Our findings support that both EC here analysed displayed evidence of continuous circulating reservoir’s reseeding and ongoing evolution of some subpopulation within the proviral quasispecies.

## SUBJECTS AND METHODS

*Study subjects* - In this study we include two EC (EEC36 and PEC38), defined as subjects infected with HIV-1 for at least five years that maintained undetectable RNA viral loads in most (> 70%) determinations without ART, harbouring heterogeneous HIV-1 proviral populations as demonstrated in a previous study of our group.^12^ Individuals has been followed-up at the Instituto Nacional de Infectologia Evandro Chagas (INI) from Rio de Janeiro (Brazil) and provided written informed consent documents approved by the INI Institutional Review Board (Addendum 049/2010) and the Brazilian National Human Research Ethics Committee (CONEP 14430/2011). The procedures followed were in accordance with the Helsinki Declaration of 1975, as revised in 1983.

Subject EEC36 is a 46-year-old woman, heterosexual, that was diagnosed with HIV-1 in 2010 and was classified in a subgroup of ebbing elite controllers (EEC) because displayed occasional and transient episodes (≤ 30% of frequency) of detectable low-level viremia (51-2,000 copies/mL) during follow-up. Subject PEC38 is also a 46-year-old woman (undisclosed exposure category) who was diagnosed with HIV-1 in 2011 and was classified as a persistent elite controller (PEC) because presents 100% of viral load measurements below the detection limit (< 40-50 copies/mL) of the available commercial assays. None of the patients had genetic factors (HLA-B and Δ32 CCR5 gene) associated with HIV-1 protection.^22^ Longitudinal analyses of proviral quasispecies over a period of ~ 6 years (2013-2019) were performed by combining the HIV-1 *env* gene sequences from our previous study^12^ and new sequences generated in this study at two visits of follow-up.

*CD4* ^*+*^ *T cell counts and plasma HIV-1 RNA quantification* - Absolute CD4^+^ T cell counts were obtained using the MultiTest TruCount-kit and the MultiSet software on a FACSCalibur flow cytometer (BD Biosciences San Jose, CA). Plasma viral load (VL) were measured according to the Brazilian Ministry of Health guidelines, with methodologies being updated overtime to improve sensitivity: the Versant HIV-1 3.0 RNA assay (bDNA 3.0, Siemens, Tarrytown, NY, limit of detection: 50 copies/mL) from 2007 to 2013, and the Abbott RealTime HIV-1 assay (Abbott Laboratories, Wiesbaden, Germany, limit of detection: 40 copies/mL) from 2013 to until now.

*Genomic DNA isolation and single genome amplification (SGA) and sequencing* - A total of 1×10^7^ cryopreserved PBMCs were thawed, washed, and immediately after, the total genomic DNA was isolated with the addition of the DNAzol^®^ Reagent (Invitrogen, USA) under conditions recommended by the manufacturers. The isolated DNA was eluted in 100 µL DNase-free water and stored at -20ºC. SGA and sequencing of DNA *env* sequences from PBMC was performed by limiting dilution nested polymerase chain reaction (PCR) using conditions previously described.^12^ The PCR products were sequenced using the ABI BigDye Terminator v.3.1 reaction Kit (Applied Biosystems, Foster City, CA) in an ABI PRISM 3100 automated sequencer (Applied Biosystem). Chromatograms were assembled into contigs using the SeqMan Pro 11 software (DNASTAR Inc., Madison, WI). Sequences resulting from chromatograms with double peaks or showing APOBEC3G/F mediated hypermutation as determined using Hypermut software^23^ were discarded.

The dataset composed of proviral *env* sequences from each individual’s three follow-up time points was submitted to the ElimDupes tool, the online Los Alamos HIV platform (https://www.hiv.lanl.gov/content/sequence/elimdupesv2/elimdupes.html) for identification of unique proviral DNA sequences and additionally we combined the results of the distance matrix performed in the MEGA 11 to confirm these unique sequences. A new dataset composed only of these unique sequences was also later used in viral evolution analysis.

*Sequence analysis: HIV-1 subtyping, analyses of viral diversity and divergence and N-glycosylation sites* - DNA viral *env* sequences were aligned with HIV-1 subtype reference sequences using ClustalW and then manually edited, yielding a final alignment covering positions 7008-7650 relative to the HXB2 reference genome (Genbank accession number: K03455.1). Maximum-likelihood (ML) phylogenetic trees were reconstructed with the PhyML 3.0 program^24^ using the most appropriate nucleotide substitution model (GTR+I+G) selected using program jModeltest v. 3.7,^25^ the sub-tree pruning & re-grafting (SPR) branch swapping heuristic tree search algorithm, and the approximate likelihood-ratio test (aLRT)^26^ for branch support.

**Fig. 1:**
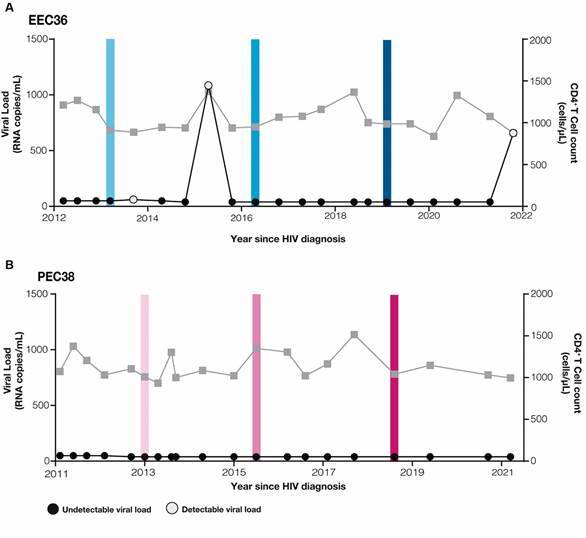
clinical follow-up from ebbing elite controllers (EEC)36 (A) and persistent elite controller (PEC)38 (B) individuals. Plasma human immunodeficiency virus (HIV) RNA viral load (copies/mL, circles) and CD4^+^ T cell counts (cells/µL, squares) since HIV diagnosis are shown on the left and the right Y-axis, respectively. Coloured shaded areas indicate the three follow-up time points that were selected for DNA quasispecies analysis.

The first dataset analysed comprised all proviral sequences from the EEC36 and PEC38 subjects (including the hypermutated sequences), combined with the HIV-1 subtype reference sequences [Supplementary data (Fig. 1)]. The complexity of proviral quasispecies at each time point was characterised using two indices after excluding hypermutated sequences: the mean nucleotide diversity (π) that measures the average number of nucleotide differences between any two sequences of the quasispecies, and the normalised Shannon entropy (H_SN_) that provides a measure of haplotype (mutant) frequencies. The π was calculated using MEGA 11^27^ and the H_SN_ was calculated by using the R package, Vegan,^28^ after rarefaction of samples to the small sample size (n = 10) for bias correction of sample size differences^29^ as described previously.^12^

To evaluate intra-host viral divergence, we first verify the temporal structure of the ML phylogenetic trees reconstructed using all sequences, all unique sequences or specific subclades of unique sequences from each patient by performing a linear regression analysis of the root-to-tip distances against sampling time using program TempEst.^30^ The intra-host viral evolutionary (divergence) rate was then directly estimated from the sampling date of the sequences for those datasets with a good temporal structure using program BEAST v1.10.^31^ The evolutionary process was estimated using the HKY + Γ4 nucleotide substitution model, a relaxed uncorrelated lognormal molecular clock model, and the parametric constant coalescent model. A continuous-time Markov chain (CTMC) rate reference prior was employed in the evolutionary rate in all datasets. We also evaluate the number of potential N-glycosylation sites (PNGSs) in *env* sequences using the online Los Alamos HIV platform (http://www.hiv.lanl.gov/content/sequence/GLYCOSITE/glycosite.html).^32^

*Statistical analysis* - The Mann-Whitney U-test was used to compare the mean number of PNGSs between viral clades. Tests were two-sided, and p-values ≤ 0.05 were considered as significant. Graphics and statistical analysis were performed using GraphPad v6 (Prism Software, United States).

*Availability of data* - The sequences from the 2016 and 2015 visits for EEC36 and PEC38, respectively, are part of a previous study and have been deposited in GenBank^®^ under accession numbers KY852611-KY852631 for EEC36 and KY852692-KY852706 for PEC38. The new HIV-1 sequences generated during the current study (2013 and 2019 visits for EEC36 and 2013 and 2019 visits for PEC38) were also deposited in GenBank^®^ under accession numbers OP799403 - OP799502.

## RESULTS

**Fig. 2:**
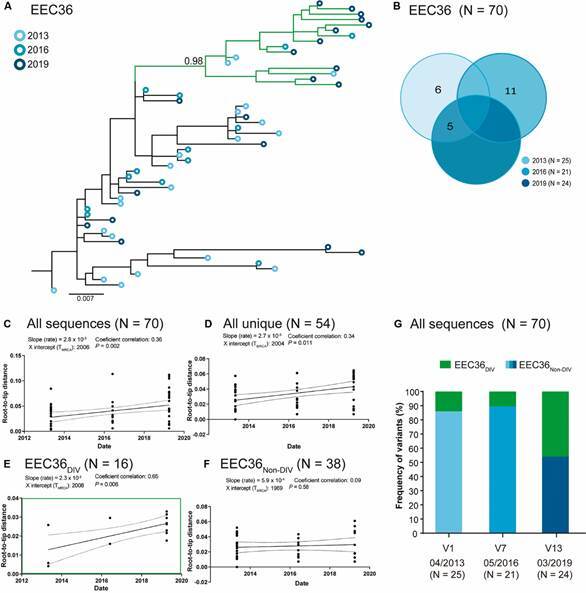
human immunodeficiency virus (HIV)-1 *env* sequences detected during the six years follow-up of subject ebbing elite controllers (EEC)36. (A) Longitudinal analysis of HIV-1 peripheral blood mononuclear cell (PBMC)-associated DNA (circles) *env* sequences obtained between 2013 and 2019. Circles in the tips of the maximum-likelihood (ML) phylogenetic tree are coloured according to the visit, as shown in the legend at the upper left corner. The green branches indicate the monophyletic subclade EEC36_DIV_ with the best temporal structure. Horizontal branch lengths are proportional to the bar at the bottom indicating nucleotide substitutions per site. The approximate likelihood-ratio test (aLRT) support is shown for EEC36_DIV_ node. (B) Venn diagrams containing the total number of identical proviral sequences detected at one (non-overlapping regions) or several (overlapping regions) time intervals during follow-up. (C-F) Plot of the root-to-tip distance of proviral sequences against sequence sampling for: (C) all proviral sequences, (D) all unique sequences, (E) unique sequences from the subclade with the best temporal structure (EEC36_DIV_), and (F) unique sequences from others subclades (EEC36_NON-DIV_). (G) Graph depicting the percentage of proviral sequences that belong to the subclades EEC36_DIV_ and EEC36_NON-DIV_ over time (month/year). DIV: evolving; NON-DIV: non-evolving.

*Clinical characteristics of patients* - Individuals EEC36 and PEC38 are two females 46 years old that have been followed up since HIV diagnoses in 2010 and 2011, respectively, maintained all (PEC38) or most (EEC36) of plasma viral load determinations below the limit of detection (< 40-50 copies/mL) without ART and CD4^+^ T cell counts within the normal range (≥ 750 cells/*μL*) (Fig. 1). Individual EEC36 displayed undetectable viremia in 18 of the 21 (86%) viral load measurements performed between 2012 and 2021 and the three blips were non-consecutive events within the range of 60-1,200 copies/mL (Fig. 1A). There were two blips between the first and second time points selected for DNA quasispecies analysis and none between the second and third timepoints. Individual PEC38 displayed undetectable viremia in all 20 viral load measurements performed between 2011 and 2021 (Fig. 1B). Longitudinal analyses revealed no evidence of immunologic progression (decline in absolute CD4^+^ T cell counts or %CD4^+^ T cells) in subjects EEC36 and PEC38 throughout follow-up [Supplementary data **(Fig. 2)]. Moreover, intra-individual variability in absolute CD4** ^+^ T cell counts in subjects EEC36 [coefficient of variation (CV) = 15.7%] and PEC38 (CV = 13.8%) were comparable to those observed during long-term (> 5 years) follow-up of ART-suppressed HIV-infected individuals (13.8-20.1%).^33^ ^,^ ^34^

*Diversity and complexity of HIV DNA quasispecies* - To measure the diversity of the PBMC-associated HIV DNA quasispecies in patients EEC36 and PEC38, we performed single genome amplification of the *env* gene by limiting dilution PCR at three time points during a period of six years between 2013 and 2019 (Fig. 1). A total of 50 new *env* sequences obtained from patient EEC36 at 2013 and 2019 were combined with 21 *env* sequences previously recovered at 2016, and a total of 51 new *env* sequences obtained from patient PEC38 at 2013 and 2019 were combined with 15 *env* sequences previously obtained at 2015. The 71 and 66 *env* sequences recovered from individuals EEC36 and PEC38, respectively, were next aligned with HIV-1 subtype reference sequences and subjected to ML phylogenetic analysis. This analysis confirmed that all *env* sequences from subjects EEC36 and PEC38 clustered in highly supported (aLRT = 100%) monophyletic groups within subtype B and subtype A1 [Supplementary data (Fig. 1)], respectively, confirming that these patients were infected by a single subtype B or A1 variant through the follow-up period. One sequence from 2013 and one from 2019 from subjects EEC36 and PEC38, respectively, were classified as hypermutated and excluded from subsequent analyses. Analyses of *env* sequences obtained at each time point revealed that subjects EEC36 and PEC 38 displayed quite diverse (π ≥ 1.9%) proviral quasispecies at all time points analysed (Table I). Although mean diversity of proviral quasispecies in subject PEC38 (π = 3.9 - 4.1%) was higher than in subject EEC36 (π = 1.9 - 3.7%), the overall complexity of proviral quasispecies in subject EEC36 (H_SN_ = 0.81 - 0.98) tend to be higher than in subject PEC38 (H_SN_ = 0.80 - 0.87) (Table I).

**TABLE I:** Virological characteristics of subjects EEC36 and PEC38

Patient ID	Visit (month/year)	RNA load (cp/mL)	Subtype	π	H_SN_	Total sequences
EEC36	V1 (Apr 2013)	< 40	B	2.4%	0.97	25
	V7 (May 2016)	< 40		1.9%	0.81	21
	V13 (Mar 2019)	< 40		3.7%	0.98	24
PEC38	V1 (Jun 2013)	< 40	A1	4.0%	0.86	16
	V4 (Dec 2015)	< 40		3.9%	0.80	15
	V9 (Jan 2019)	< 40		4.1%	0.87	34

EEC: ebbing elite controllers; PEC: persistent elite controller; H_SN_: the normalised Shannon entropy; π: the mean nucleotide diversity.

**Fig. 3:**
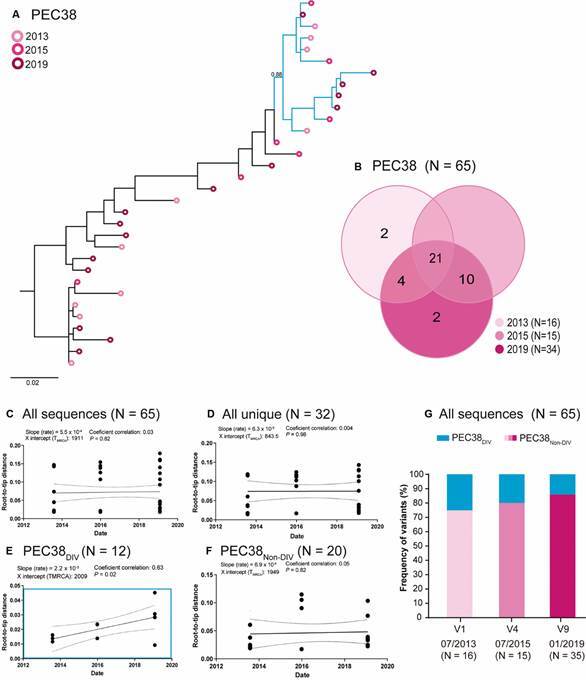
human immunodeficiency virus (HIV)-1 *env* sequences detected during the six years follow-up of subject persistent elite controller (PEC)38. (A) Longitudinal analysis of HIV-1 peripheral blood mononuclear cell (PBMC)-associated DNA (circles) *env* sequences obtained between 2013 and 2019. Circles in the tips of the maximum-likelihood (ML) phylogenetic tree are coloured according to the visit, as shown in the legend at the upper left corner. The blue branches indicate the monophyletic subclade PEC38DIV with the best temporal structure. Horizontal branch lengths are proportional to the bar at the bottom indicating nucleotide substitutions per site. The approximate likelihood-ratio test (aLRT) support is shown for PEC38_DIV_ node. (B) Venn diagrams containing the total number of identical proviral sequences detected at one (non-overlapping regions) or several (overlapping regions) time intervals during the follow-up. (C-F) Plot of the root-to-tip distance of proviral sequences against sequence sampling time for: (C) all proviral sequences, (D) all unique sequences, (E) unique sequences from the subclade with the best temporal structure (PEC38_DIV_), and (F) unique sequences from other subclades (PEC38_NON-DIV_). (G) Graph depicting the percentage of proviral sequences that belong to the subclades PEC38_DIV_ and PEC38_NON-DIV_ over time. DIV: evolving; NON-DIV: non-evolving.

*Evolution of HIV DNA quasispecies* - In both individuals viral quasispecies, *env* sequences sampled at different time points were highly intermingled in the ML phylogenetic tree (Figs 2A-3A). To obtain a clearer picture of the proviral dynamics over time, we first compared the number of identical DNA viral sequences that could be used as surrogate markers of “proviral clones” maintained by clonal expansion of infected cells. Next, we analyse the temporal structure (root-to-tip distance against sequence sampling time) as a surrogate marker of viral divergence. Linear regression was used to determine if slopes were significantly different (p < 0.05) from zero. We estimate the temporal structure of different datasets including: (i) all quasispecies sequences; (ii) all unique sequences by retaining only one sequence (one of the earliest) from each proviral clone to reduce the impact of latency on intra-host evolutionary divergence; (iii) highly supported (aLRT ≥ 0.85) subclades comprising at least 10 sequences covering the whole period of study; and (iv) unique sequence datasets without sequences from divergent subclade.

In subject EEC36, identical sequences represent a minor fraction of the whole quasispecies (31%, 22/70) and only a few of them persist across different time points (Fig. 2B), indicating a substantial renewal of circulating viral reservoir over time. Consistent with this observation, we detected a significant increase in the genetic divergence for datasets including all viral sequences (Fig. 2C) and all unique sequences (Fig. 2D). In addition, we also identified three highly supported subclades that showed a significant increase in genetic divergence over time [Supplementary data (Fig. 3)]. The subclade with the best temporal structure (highest coefficient of correlation), designated as EEC36_DIV_, comprises 30% (n = 16) of unique sequences and displayed a similar divergence rate (slope) as datasets comprising all viral sequences (n = 70) and all unique sequences (n = 54) (Fig. 2E). Importantly, when the subclade EEC36_DIV_ was removed, the remaining unique sequences from other subclades (EEC36_NON-DIV_) displayed no significant temporal structure (Fig. 2F), supporting that subclade EEC36_DIV_ essentially explained the temporal structure of datasets including all viral sequences and all unique sequences. Of note, the subclade EEC36_DIV_ becomes increasingly predominant over time, rising from 12% of quasispecies sequences in 2013 to 46% in 2019 (Fig. 2G).

**Fig. 4:**
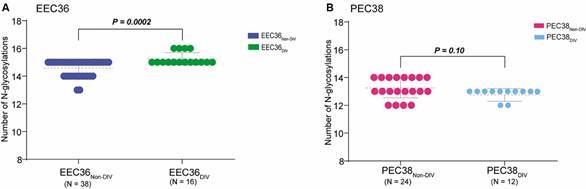
analyses of N-linked glycosylation in the *env* gp120 C2-C4 region of viral sequences from subjects ebbing elite controllers (EEC)36 and persistent elite controller (PEC)38. Dot plots with the numbers of estimated potential N-glycosylation sites (PNGSs) in *env* sequences from EEC36_NON-DIV_ and EEC36_DIV_ (A) and PEC38_NON-DIV_ and PEC38_DIV_ (B). Horizontal lines represent the mean and standard deviation. Two-tailed Mann-Whitney U tests were used. DIV: evolving; NON-DIV: non-evolving.

In subject PEC38, the overall proportion of identical DNA viral sequences (49%, 32/65) and of proviral clones detected in at least two (21%, 14/65) or three (32%, 21/65) time points was higher than in subject EEC36 (Fig. 3B), supporting a more limited renewal of the viral reservoir. Consistent with this observation, we found no evidence of measurable divergence over time for datasets including all viral sequences (Fig. 3C) and all unique sequences (Fig. 3D). We also failed to detect measurable divergence in most highly supported subclades, with the exception of two subclades that displayed a significant increase in the genetic divergence over time [Supplementary data (Fig. 4)]. The subclade with the best temporal structure (highest coefficient of correlation), designated as PEC38_DIV_, comprised 38% (n = 12) of unique sequences and displayed a higher divergence rate (slope) than datasets comprising all viral sequences and all unique sequences (Fig. 3E). As expected, when the subclade PEC38_DIV_ is removed, the remaining unique sequences (PEC38_NON-DIV_) display no significant temporal structure (Fig. 3F). The frequency of the subclade PEC38_DIV_ tends to decrease over time, ranging from 25% in 2013 to 15% in 2019 (Fig. 3G).

To estimate the intra-host HIV-1 *env* divergence rate in subjects EEC36 and PEC38, we performed a Bayesian molecular clock analysis of datasets with significant temporal structure and that only comprise unique sequences. The Bayesian molecular clock analyses estimate an overall mean intra-host *env* evolutionary rate of: 1.5 × 10^-3^ subst/site/year (95% HPD: 5.5 × 10^-4^ - 2.9 × 10^-3^ subst/site/year) for all unique sequences of subject EEC36, 5.0 × 10^-3^ subst/site/year (95% HPD: 1.3 × 10^-3^ - 1.6 × 10^-2^ subst/site/year) for subclade EEC36_DIV_, and 2.9 x 10^-3^ subst/site/year (95% HPD: 5.7 × 10^-7^ - 1.7 × 10^-2^ subst/site/year) for subclade PEC38_DIV_. These analyses support that the subclades EEC36_DIV_ and PEC38_DIV_ have been evolving at a slow but measurable rate over long-term follow-up. Although the median evolutionary rate of subclade EEC36_DIV_ was about two times higher than that estimated from all unique sequences, consistent with the better temporal structure of the sub-clade respect to other sequences from quasispecies, the HPD intervals of both estimates displayed substantial overlap and no definitive conclusions could be obtained.

To understand the potential impact of *env* changes on virus phenotype, we compared the diversity and number of PNGSs in *env* sequences from evolving and non-evolving (ancestral) subpopulations from each subject. We observed a significant (p = 0.0002) increase in the mean number of PNGSs in the evolving (EEC36_DIV_) respect to non-evolving (EEC36_NON-DIV_) proviruses of subject EEC36 (Fig. 4A). While PNGSs at the C2-C3 region were quite conserved, with positive signatures for neutralisation detected as the most common variant, PNGSs at the V4-C4 region were more variable (Table II). The PNGSs N406 and N413 were only detected in EEC36_NON-DIV_ variants (> 90%), while the PNGSs N411 and N442 were only detected among EEC36_DIV_ variants (75-100%). We do not observe significant (p = 0.10) difference in the mean number of PNGSs between evolving (PEC38_DIV_) and non-evolving (PEC38_NON-DIV_) proviruses of subject PEC38 (Fig. 4B), but we observed several important variations among sites (Table II). The PNGSs N289, N295, N401 and N463 were much more frequent among PEC38_DIV_ variants (83-100%) respect to PEC38_NON-DIV_ variants (35-70%), the PNGSs N355 and N466 were less frequently detected among PEC38_DIV_ variants (42-58%) respect to PEC38_NON-DIV_ variants (70-95%), and the PNGS N363, N399 and N462 were only detected among PEC38_NON-DIV_ variants (25-70%).

**TABLE II:** Proportion of sequences in evolving (DIV) and non-evolving (NON-DIV) and proviral subpopulation of subjects EEC36 and PEC38 with mutations at potential N-glycosylation sites (PNGSs) that may modulate the neutralisation phenotype and/or viral infectivity

Position (HXB2)	Region	EEC36_NON-DIV_	EEC36_DIV_	PEC38_NON-DIV_	PEC38_DIV_
276	C2	97%	100%	100%	100%
289	C2	100%	100%	65%	92%
295	C2	100%	100%	70%	100%
301	V3	100%	100%	100%	100%
332	C3	100%	100%	100%	100%
339	C3	94%	100%	---	---
355	C3	100%	100%	70%	58%
362	C3	81%	100%	---	---
363	C3	---	---	25%	--
386	V4	100%	100%	90%	100%
392	V4	100%	100%	100%	100%
397	V4	92%	75%	---	---
399	V4	---	---	55%	---
401	V4	---	---	45%	83%
406	V4	92%	---	100%	100%
411	V4	---	75%	---	---
413	V4	100%	---	---	---
442	C4	---	100%	100%	100%
448	C4	100%	100%	95%	100%
461	V5	97%	100%	---	---
462	V5	---	---	70%	---
463	V5	---	---	35%	83%
466	V5	---	---	95%	42%

EEC: ebbing elite controllers; PEC: persistent elite controller.

## DISCUSSION

In this study, we investigate the long-term (> 5 years) HIV evolution in two EC with relatively diverse circulating proviral reservoirs, in the setting of persistent undetectable viremia (subject PEC38) and occasional blips (subject EEC36) and no evidence of immunologic progression. We detected genetic markers of ongoing evolution and continuous renewal in the PBMCs-associated proviral sequences of both EC during follow-up. HIV-infected PBMCs from both EC comprised a significant fraction of identical (probably clonally expanded) archival proviruses and unique evolving proviruses. We hypothesise that subjects EEC36 and PEC38 may display an efficient mechanism of suppression of extrafollicular HIV replication that maintains plasma viremia below detectable levels, but fails to fully suppress viral replication (and viral evolution) within lymphoid tissues.^9^ ^,^ ^35^ Continuous trafficking of newly infected cells from lymphoid tissues to peripheral blood resulted in a slow, but measurable, long-term reseeding of the circulating reservoir.^9^

The detection of unique evolving proviruses in circulating reservoirs was previously described after long-term follow-up (> 10 years) of another EC (subject EEC42) from our cohort that displayed quite frequent blips (30% of viral load determinations above the detection limit of commercial assays).^12^ The present study demonstrates that long-term ongoing proviral evolution may also occur in EC with occasional blips (EEC36, 14% of viral load determinations) and even in EC with persistent undetectable viremia (PEC38), but with different rate. The overall proportion of unique proviral sequences in subject PEC38 (49%) was lower than in subjects with blips (70 - 80%). Furthermore, we detected measurable divergence in the whole proviral quasispecies of subjects EEC36 and EEC42, but not in subject PEC38. These findings suggest that the relative contribution of clonally expanded archival proviruses *vs* unique evolving proviruses to the long-term landscape of the circulating reservoir in EC may vary among individuals according to the levels of cumulative residual viral replication and of trafficking of newly infected cells between tissue compartments.

Previous studies detected evidence of ongoing HIV-1 evolution in plasma sequences of some EC, but failed to detect measurable evolution of PBMCs-associated proviral sequences.^9^ ^,^ ^14^ ^,^ ^15^ ^,^ ^20^ ^,^ ^36^ ^-^ ^38^ Detection of ongoing evolution of circulating reservoirs in EC may have been hampered in previous studies by the significant fraction of latent archival proviruses that compose the quasispecies combined with the narrow sampling interval (< 5 years) and/or the few (n < 10) proviral sequences obtained at each time-point. Ongoing evolution in the peripheral DNA viral compartment of EC may become only apparent when: (i) proviral samples are obtained over wide sampling intervals (> 5 years); (ii) a substantial number of proviral clones (n > 15) are amplified at each point; and (iii) the analyses are focused on unique sequences from specific subclades that are probably replicating instead of sequences from the bulk proviral quasispecies, as observed in subject PEC38.

Divergent patterns of HIV persistence were described in the setting of fully suppressive ART and viremic controllers (VC). While several studies reported no evidence of ongoing evolution in PBMCs-associated DNA viral sequences during ART,^39^ ^,^ ^40^ ^,^ ^41^ ^,^ ^42^ ^,^ ^43^ one study described continued infections of cells in lymphoid tissue sanctuary sites and common trafficking to bloodstream that resulted in the replenish and ongoing evolution of PBMCs-associated proviral reservoir during ART.^21^ Another study with four VC showed that two participants presented proviral sequences that integrated recently, while proviruses recovered from the other two participants dated to time points spread throughout infection, consistent with different rates of reservoir turnover.^44^ These findings point to the probable coexistence of different mechanisms of HIV persistence (clonal amplification and ongoing replication) in EC, VC and treated subjects and further support that ART may provide and additional layer of protection to some EC, by further reducing the level of residual HIV replication and ongoing viral evolution.^45^ ^,^ ^46^ ^,^ ^47^ ^,^ ^48^

Changes in HIV-1 *env* glycosylation pattern represents an important mechanism of escape from broadly neutralising antibodies (bNAb)^49^ ^,^ ^50^ and the number of PNGSs in *env* typically increased over the first years of infection, as the virus became more resistant to autologous Nab.^51^ ^,^ ^52^ ^,^ ^53^ Evolving variants display a higher frequency of PNGSs than ancestral ones^54^ ^,^ ^55^ and proviruses of EC subjects showed less glycosylated and less-functional *env* sequences than proviruses from progressors’ individuals.^55^ ^,^ ^56^ Of note, we detected a significantly higher mean number of PNGSs in proviruses of the evolving clade (EEC36_DIV_) than in other proviruses of subject EEC36, suggesting that this clade may display a higher resistance to bNab and/or better *env* functionality, consistent with its increasing prevalence over time. This result, however, should be interpreted with caution as many PNGSs increase sensitivity to bNAb or may have opposite effects depending on the bNAb class. Among PNGSs over-represented in EEC36_DIV_ proviruses, site N362 was correlated with sensitivity to V3/CD4bs bNab and site N442 was correlated with sensitivity to V3 bNab and resistance to CD4bs bNab.^57^ The PNGS N413, which was under-represented among EEC36_DIV_ variants, was correlated with sensitivity to V3 bNab and resistance to MPER bNab.^58^

Although the mean number of PNGSs did not vary significantly between evolving (PEC38_DIV_) and other proviruses of subject PEC38, we observed differences at several key positions. Among PNGSs over-represented in PEC38_DIV_ proviruses, sites N295 and N289 were correlated with sensitivity and resistance to V3 bNab, respectively.^59^ Notably, preservation of the PNGS N289 was also important for high viral infectivity.^57^ None of PNGSs under-represented in PEC38_DIV_ proviruses (N355, N363, N399, N462 and N466) were correlated with changing sensitivity to bNAb; but nearby PNGSs were associated with sensitivity to bNAb (N362) or autologous NAb in HIV controllers (N397 and N460).^53^ These findings suggest that PEC38_DIV_ proviruses may display differential resistance to bNab and/or infectivity than other proviral variants. Despite these potential advantages, the frequency of PEC38_DIV_ proviruses decreased over time, supporting that the host immune response in this subject was more efficient to contain the refuelling of circulating reservoirs by evolving variants than in subject EEC36.

Functionality of the Env protein could be a relevant determinant of *in vivo* viral replication control and pathogenesis^60^ and one study described that high level of HIV proviral *env* diversity in EC is associated with future loss of natural virologic control.^11^ Other studies, however, suggests that *env* or *pol* genetic diversity of the HIV-1 proviral reservoir is not an accurate biomarker for the prediction of virologic breakthrough in EC.^61^ ^,^ ^62^ Indeed, subjects EEC36 and PEC38 maintained virological and immunological control during follow-up despite evidence of ongoing proviral evolution. Similarly, other studies described that EC maintains virologic control despite selection of immune escape mutations in plasma viral sequences.^9^ ^,^ ^10^ ^,^ ^13^ ^-^ ^15^ ^,^ ^20^ ^,^ ^36^ ^-^ ^38^ ^,^ ^63^ ^,^ ^64^ These findings suggest that host immunity in some EC can efficiently control HIV-1 infection without completely blocking viral replication and evolution, but we cannot rule out the possibility of long-term immunological attrition due to persistent low levels of viral replication and evolution in those subjects.

Our study has some limitations. First, we cannot exclude that some proviral sequences described as unique were not detected at multiple time-points due to the limited sampling size or that they were persistently detected in lymphoid tissues but only recirculate in blood at some specific time points. Second, we do not explore the evolution of RNA viral sequences in the plasma compartment of EC subjects, and we thus cannot confirm the hypothesis that divergent (and evolving) proviral clades identified in our analyses correspond to actively replicating viral variants. Third, although the changes in the HIV-1 *env* glycosylation pattern between evolving and non-evolving proviruses are consistent with differential resistance to neutralisation and/or *env* functionality, we do not experimentally assess the phenotype of different viral variants. Finally, our findings need to be confirmed in larger cohorts of EC with diverse proviral populations.

In summary, this study confirms that the relatively high HIV-1 *env* diversity detected in the circulating proviral population of some EC resulted from a process of ongoing viral evolution and reservoir’s reseeding combined with long-term persistence of ancestral proviruses. The ongoing viral evolution may result in the emergence of variants with altered immunological and/or virological phenotypes in EC, despite persistent virologic control. Such evolved variants may progressively replace the ancestral ones or may remain as minor variants in the circulating proviral reservoir. Continuous follow-up would be important to assess the potential association of ongoing viral evolution and reservoir replenishment with future immunologic and/or virologic breakthrough in EC and to identify those subjects that may benefit from ART.
