# Supplementary material for: Ongoing HIV-1 evolution and reservoir reseeding in two elite controllers with genetically diverse peripheral proviral quasispecies
Source: Mem Inst Oswaldo Cruz. 2023 Jun 5;118:e230066. doi: 10.1590/0074-02760230066 (PMC10292822; doi:10.1590/0074-02760230066)
Supplement: Supplementary file 1 [file 1678-8060-mioc-118-e230066-s.pdf]

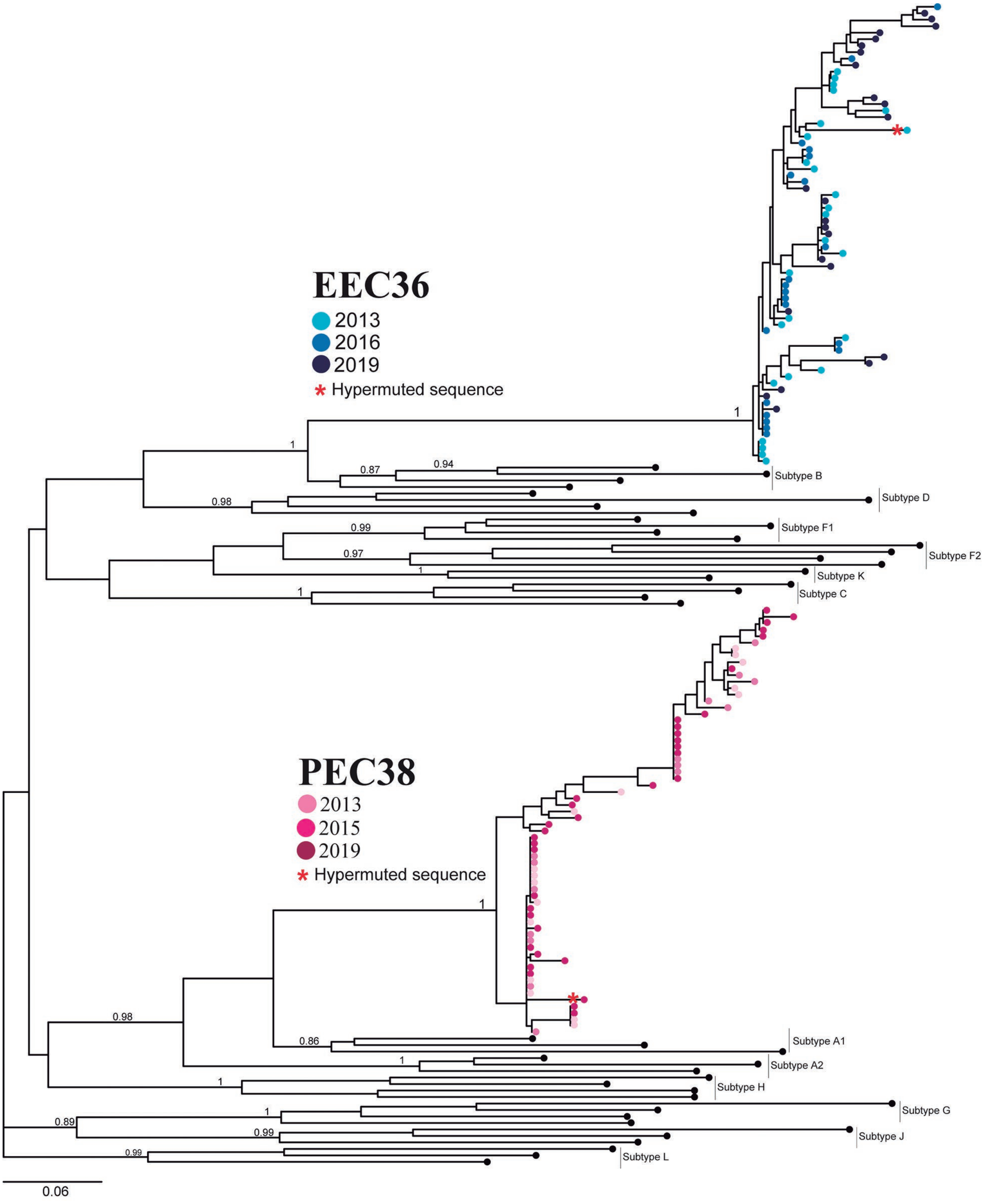


Fig. 1: maximum-likelihood (ML) tree subtype classification and contamination identification. The years of the smples for each patient re indicated in the legends and indicated at the tip circles. Human immunodeficiency virus (HIV)-1 references sequences were obtained from Los Alamos Database.

CC BY

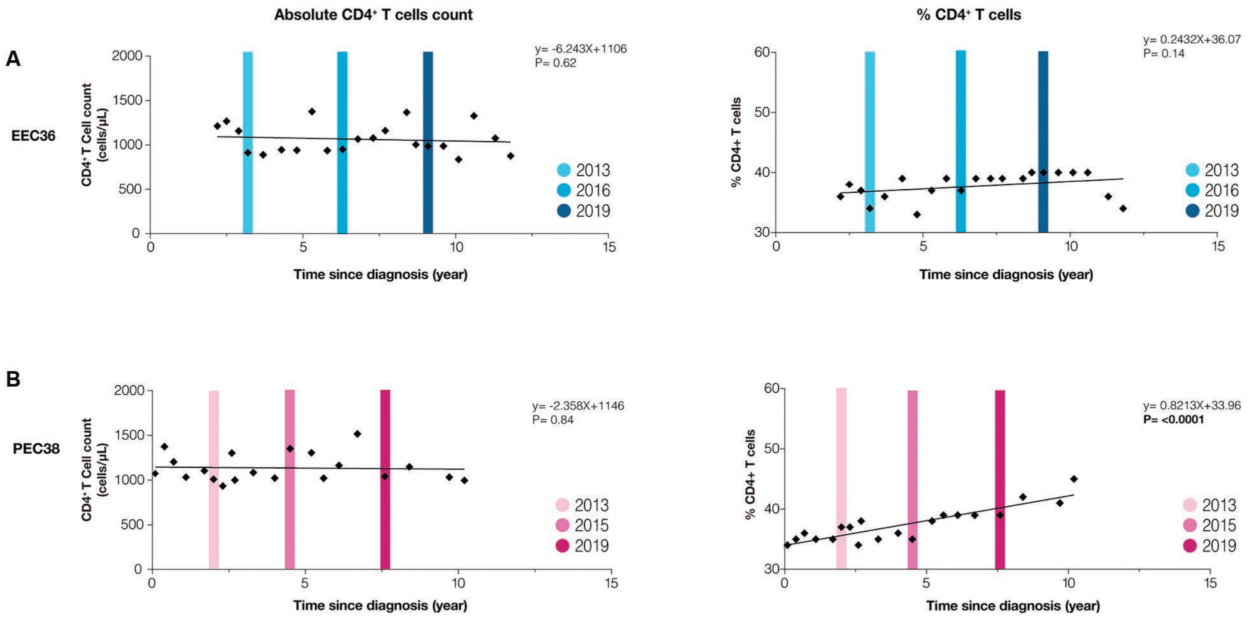


Fig. 2: longitudinal analyses of the CD4+ T cell cout (cells/µL) and the %CD4+ T cells of patients ebbing elite controllers (EEC)36 (A) and persistent elite controller (PEC)38 (B). Coloured shaded areas indicate the three follow-up time points that were selected for DNA quasispecies analysis.

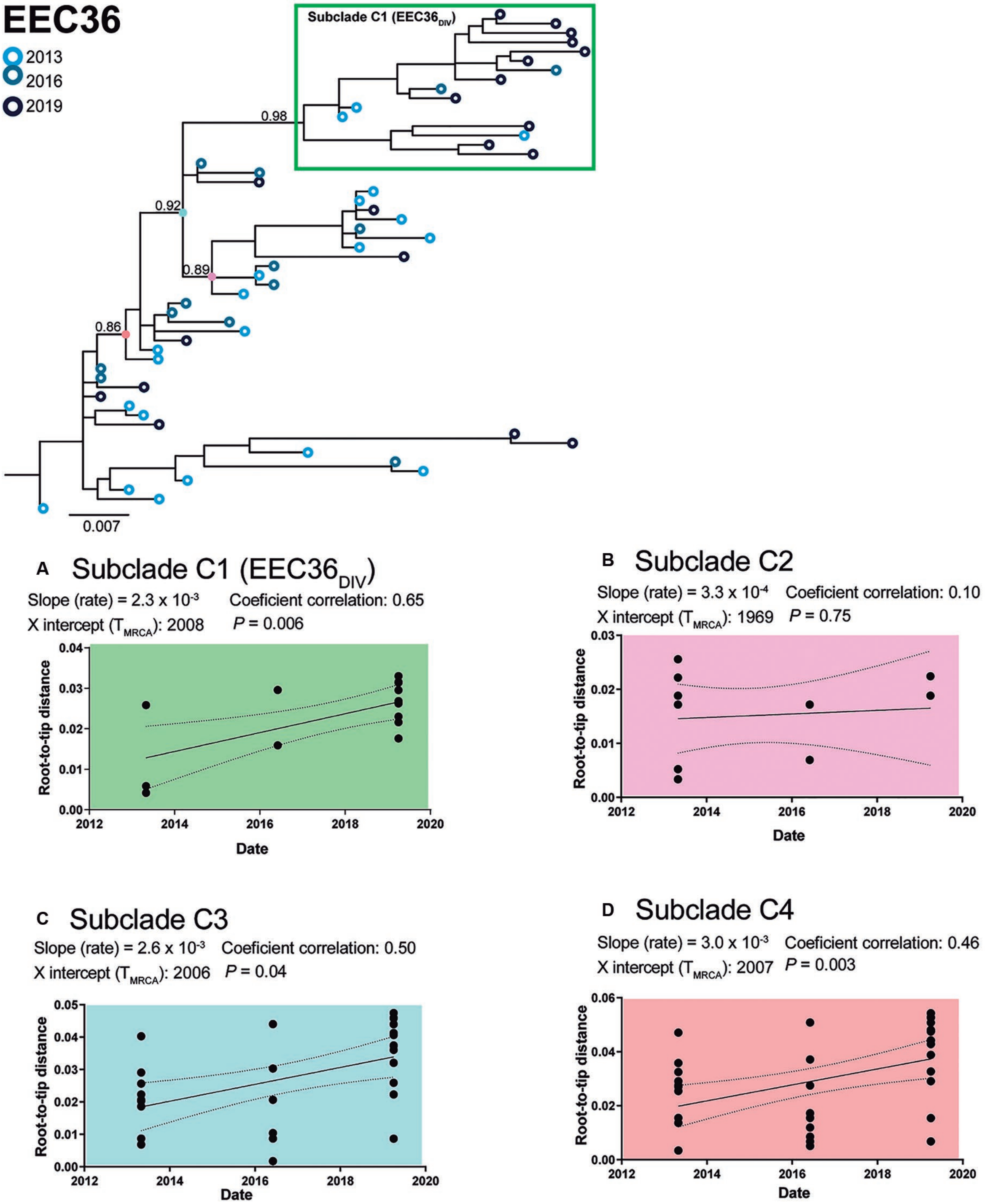


Fig. 3: maximum-likelihood (ML) phylogenetic tree of the unique human immunodeficiency virus (HIV)-1 proviral *env* sequences from ebbing elite controllers (EEC)36 was coloured according to the visit, as shown in the legend at the upper left corner. The branches inside the green rectangle indicate the monophyletic subclade $EEC36_{DIV}$ with the best temporal structure. The $EEC36_{NON-DIV}$ subclades ancestral nodes are indicated by circles with different colours. (A-D) The plot of the rot-to-tip distance of proviral sequences against sampling time for each non-divergent subclade are depicted on its corresponding colour. DIV: evolving; NON-DIV: non-evolving.

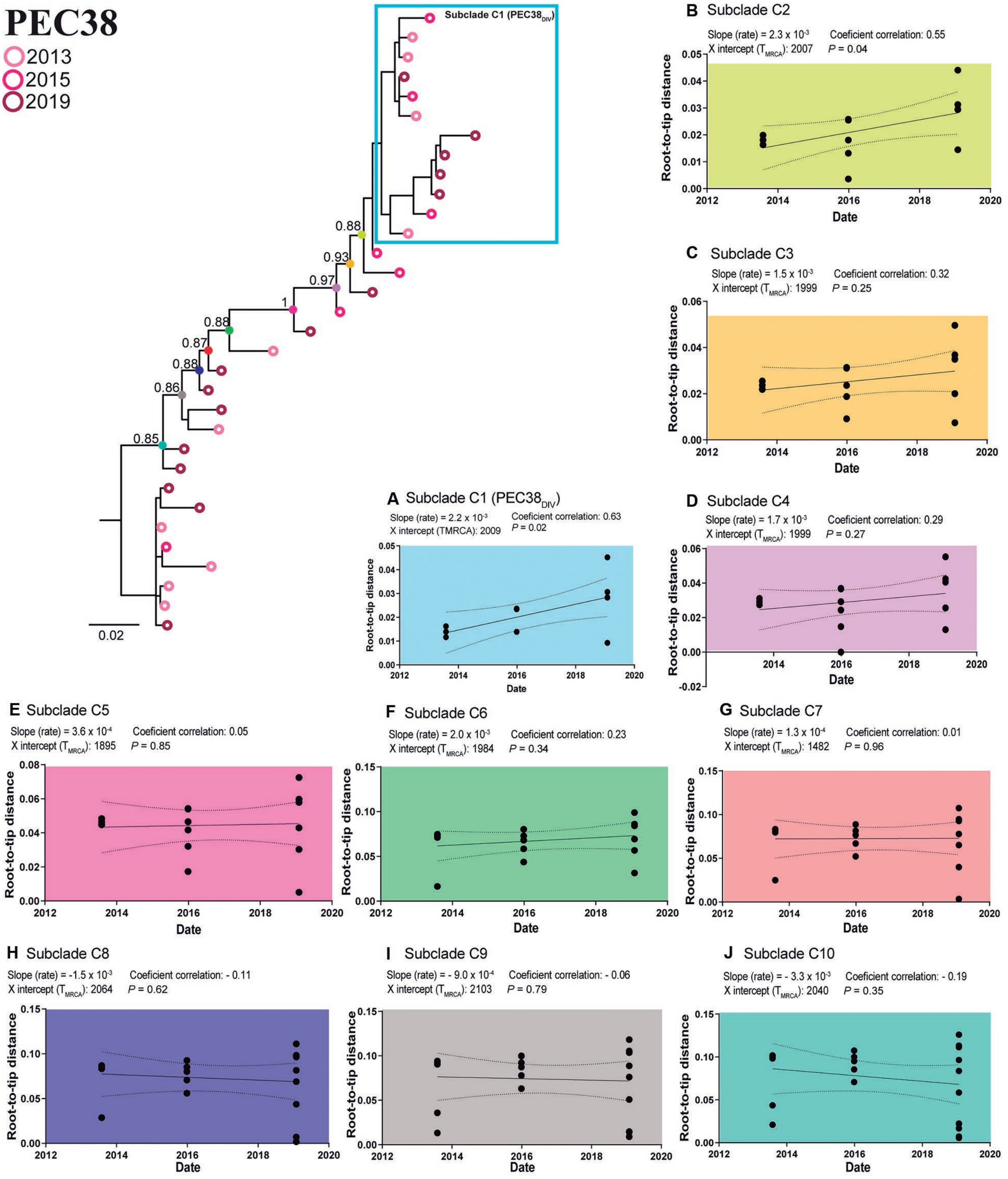


Fig. 4: maximum-likelihood (ML) phylogenetic tree of the unique human immunodeficiency virus (HIV)-1 proviral *env* sequences from persistent elite controller (PEC)38 was coloured according to the visit, as shown in the legend at the upper left corner. The branches inside the green rectangle indicate the monophyletic subclade $PEC38_{DIV}$ with the best temporal structure. The $PEC38_{NON-DIV}$ subclades ancestral nodes are indicated by circles with different colours. (A-J) The plot of the rot-to-tip distance of proviral sequences against sampling time for each non-divergent subclade are depicted on its corresponding colour. DIV: evolving; NON-DIV: non-evolving.
